# Host Cellular Protein TRAPPC6AΔ Interacts with Influenza A Virus M2 Protein and Regulates Viral Propagation by Modulating M2 Trafficking

**DOI:** 10.1128/JVI.01757-16

**Published:** 2016-12-16

**Authors:** Pengyang Zhu, Libin Liang, Xinyuan Shao, Weiyu Luo, Shuitao Jiang, Qingqing Zhao, Nan Sun, Yuhui Zhao, Junping Li, Jinguang Wang, Yuan Zhou, Jie Zhang, Guangwen Wang, Li Jiang, Hualan Chen, Chengjun Li

**Affiliations:** State Key Laboratory of Veterinary Biotechnology, Harbin Veterinary Research Institute, Chinese Academy of Agricultural Sciences, Harbin, China; University of Pittsburgh School of Medicine

**Keywords:** influenza A virus, M2, TRAPPC6AΔ, transport

## Abstract

Influenza A virus (IAV) matrix protein 2 (M2) plays multiple roles in the early and late phases of viral infection. Once synthesized, M2 is translocated to the endoplasmic reticulum (ER), travels to the Golgi apparatus, and is sorted at the *trans*-Golgi network (TGN) for transport to the apical plasma membrane, where it functions in virus budding. We hypothesized that M2 trafficking along with its secretory pathway must be finely regulated, and host factors could be involved in this process. However, no studies examining the role of host factors in M2 posttranslational transport have been reported. Here, we used a yeast two-hybrid (Y2H) system to screen for host proteins that interact with the M2 protein and identified transport protein particle complex 6A (TRAPPC6A) as a potential binding partner. We found that both TRAPPC6A and its N-terminal internal-deletion isoform, TRAPPC6A delta (TRAPPC6AΔ), interact with M2. Truncation and mutation analyses showed that the highly conserved leucine residue at position 96 of M2 is critical for mediating this interaction. The role of TRAPPC6AΔ in the viral life cycle was investigated by the knockdown of endogenous TRAPPC6AΔ with small interfering RNA (siRNA) and by generating a recombinant virus that was unable to interact with TRAPPC6A/TRAPPC6AΔ. The results indicated that TRAPPC6AΔ, through its interaction with M2, slows M2 trafficking to the apical plasma membrane, favors viral replication *in vitro*, and positively modulates virus virulence in mice.

**IMPORTANCE** The influenza A virus M2 protein regulates the trafficking of not only other proteins but also itself along the secretory pathway. However, the host factors involved in the regulation of the posttranslational transport of M2 are largely unknown. In this study, we identified TRAPPC6A and its N-terminal internal-deletion isoform, TRAPPC6AΔ, as interacting partners of M2. We found that the leucine (L) residue at position 96 of M2 is critical for mediating this interaction, which leads us to propose that the high level of conservation of 96L is a consequence of M2 adaptation to its interacting host factor TRAPPC6A/TRAPPC6AΔ. Importantly, we discovered that TRAPPC6AΔ can positively regulate viral replication *in vitro* by modulating M2 trafficking to the plasma membrane.

## INTRODUCTION

Influenza A virus (IAV) is an important zoonotic pathogen that causes significant morbidity and mortality in both humans and animals. It is a member of the Orthomyxoviridae family and has a genome composed of eight single-stranded, negative-sense RNA segments. The IAV genome encodes 10 essential proteins as well as some more recently identified accessory proteins (1–7). Matrix protein 2 (M2) is the smallest structural protein; it is synthesized from a spliced mRNA transcript of the M segment. It is a 97-amino-acid protein, comprising three distinct domains: a 24-residue ectodomain, a 19-residue transmembrane domain, and a 54-residue cytoplasmic tail (CT) domain (8, 9). The first 17 residues of the M2 CT domain form a membrane-parallel amphiphilic helix with both a hydrophilic side and a hydrophobic side (10, 11). The CT domain also contains a binding site for matrix protein 1 (M1) (12, 13). M2 associates in the membrane as a homotetramer that is stabilized by the formation of disulfide bonds between the monomers (14).

M2 plays multiple roles in the life cycle of IAV. Its tetrameric transmembrane domain forms a proton-selective ion channel that conducts protons from the acidified endosomes into the interior of the virus particle to dissociate the viral ribonucleoprotein (vRNP) complexes from M1 and the lipid bilayers, thus completing the uncoating process during virus entry (15, 16). Defects in M2 proton channel activity result in reduced virus replication in tissue culture and in mice. The antiviral drugs amantadine and rimantadine bind to the M2 proton channel, thus preventing proton conduction and inhibiting virus entry (17). The proton channel activity of M2 can also raise the pH of the *trans*-Golgi network (TGN) to prevent the premature low-pH conformational change of viral hemagglutinin (HA) during its transport to the cell surface. This function is important for highly pathogenic H7 influenza viruses, whose HA can be cleaved by ubiquitous proteases and is therefore more susceptible to low-pH-induced premature maturation (18, 19) and also essential to maintain 2009 pandemic H1N1 virus HA fusion competence during transport to the cell surface (20). In addition, the M2 proton channel activity can activate the NLRP3 inflammasome of macrophages and dendritic cells (21).

The M2 protein is also required for virus assembly and budding. M2 is positioned at the edge of the budozone when virus budding begins and induces negative membrane curvature in a cholesterol-dependent manner (22, 23), thus stabilizing the HA- and M1-induced positive membrane curvature at the budozone. During virus release, M2 localizes at the neck of the budding virion with an amphiphilic helix inserted into the membrane, induces positive membrane curvature, and finally pinches off the budding virion (22). Mutation or deletion of the M2 CT domain markedly impairs the assembly of viral proteins and genome segments into virions, filamentous virion formation, and virion release (12, 13, 22, 24–26). Moreover, the ectodomain of M2 is also important for virion incorporation (27).

Another function attributed to the M2 protein is an effect on autophagy. M2 expression during virus infection inhibits autophagosome degradation by blocking the fusion of autophagosomes and lysosomes, leading to enhanced death of virus-infected cells (28). It was recently demonstrated that M2 proton channel activity contributes to the block of autophagosome-lysosome fusion and that the effect of M2 on autophagy arrest can be inhibited by the proton channel inhibitor amantadine (29).

The transport protein particle (TRAPP) complex was the first multisubunit tethering complex to be identified and structurally characterized (30). In Saccharomyces cerevisiae, three forms of the TRAPP complex, TRAPP I, TRAPP II, and TRAPP III, have been identified (31–34). TRAPP I is essential for endoplasmic reticulum (ER)-to-Golgi protein trafficking (31), TRAPP II is important for intra-Golgi trafficking (35), and TRAPP III is involved in autophagy (32). TRAPP complexes also regulate ER-to-Golgi and intra-Golgi traffic in mammalian cells (36, 37). Transport protein particle complex 6A (TRAPPC6A) is a full-length protein of 173 amino acids. In addition to TRAPPC6A, which is also termed isoform 1, there are three other translational isoforms derived from alternative splicing of the mRNA transcript. Isoform 2, which is also termed TRAPPC6AΔ (38), is identical to full-length TRAPPC6A except for a 14-amino-acid deletion at positions 29 to 42. In contrast, isoforms 3 and 4 are 127 and 113 amino acids long, respectively, and share very little homology with TRAPPC6A due to the near-N-terminal frameshift. TRAPPC6AΔ is a subunit of TRAPP complexes. A recessive mutation in TRAPPC6AΔ (named TRAPPC6A in the original study) leads to a mosaic loss of coat pigment in mice, indicating that a defect in early secretory protein traffic caused by this mutation may interfere with melanosome biogenesis (39). TRAPPC6AΔ aggregates form plaques in the hippocampi of postmortem middle-aged normal humans and Alzheimer's disease patients, thus playing a critical role in neurodegeneration (38). However, no studies have implicated TRAPPC6A or TRAPPC6AΔ in viral life cycles.

M2 relies on the host cell machinery to fulfill its functions during the virus life cycle. However, M2 could also be a target of the host defense system. So far, only a few host cellular proteins have been reported to physically interact with M2 (40–43). Therefore, most of the M2-interacting partners have yet to be identified, and their functions remain to be elucidated. In the present study, we used a yeast two-hybrid (Y2H) system to screen for host proteins that interact with the M2 protein and identified TRAPPC6A to be a potential binding partner. By using coimmunoprecipitation (co-IP) and confocal microscopy, we demonstrated that both TRAPPC6A and its N-terminal internal-deletion isoform, TRAPPC6AΔ, interact with M2. We discovered that the highly conserved leucine residue at position 96 of M2 is indispensable for this interaction. Our results demonstrate that TRAPPC6AΔ is involved in the posttranslational trafficking of M2 to the plasma membrane, favors virus growth *in vitro*, and positively modulates virus virulence in mice.

## RESULTS

### Identification of cellular TRAPPC6A as a novel M2-interacting protein.

**FIG 1:**
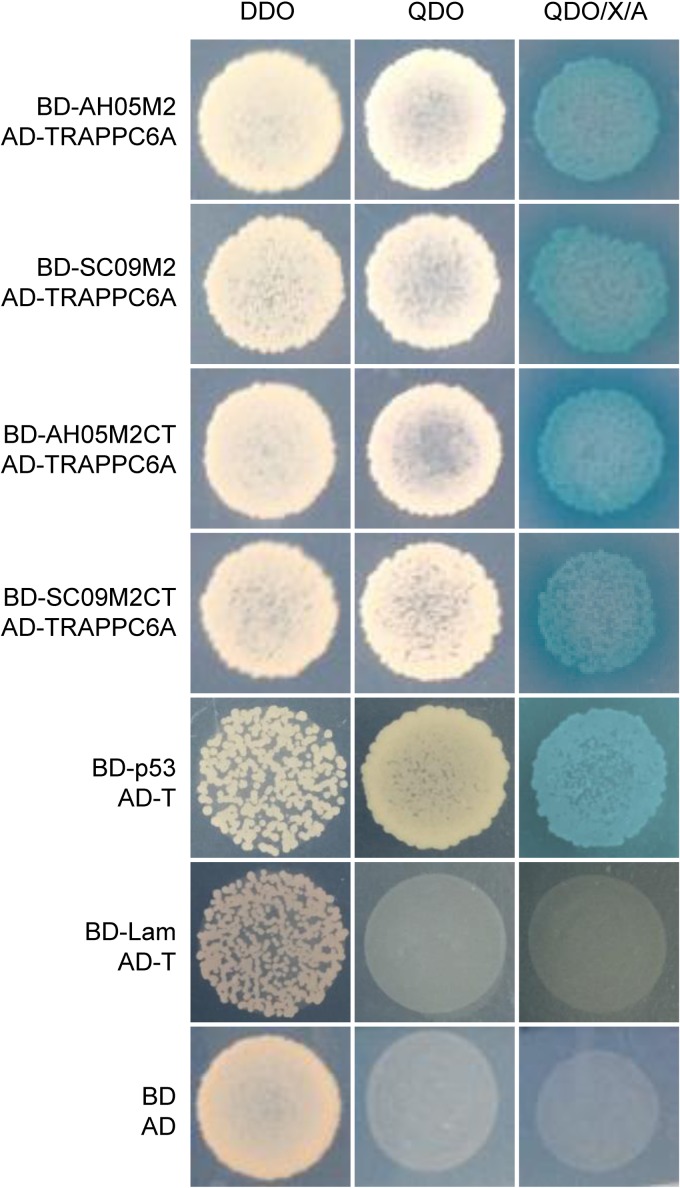
Protein-protein interaction of IAV M2 with TRAPPC6A in the Y2H system. Yeast strain Y2HGold was cotransformed with one of the bait plasmids (BD-AH05M2, BD-SC09M2, BD-AH05M2CT, or BD-SC09M2CT), together with the prey plasmid AD-TRAPPC6A, which encodes TRAPPC6A fused to the Gal4 activation domain. Positive protein-protein interactions are indicated by the appearance of blue colonies in the presence of X-α-Gal. Cotransformation of pGBKT7-53 encoding the Gal4 DNA-BD fused with murine p53 (BD-p53) and pGADT7-T encoding the Gal4 activation domain fused with the simian virus 40 large T antigen (AD-T) served as a positive control, since p53 and large T antigen are known to interact in the Y2H system. Cotransformation of pGBKT7-Lam, which encodes the Gal4-BD fused with human lamin C (BD-Lam), and AD-T served as a negative control, since lamin C does not form complexes or interact with most other proteins. DDO, SD/−Leu/−Trp; QDO, SD/−Ade/−His/−Leu/−Trp; QDO/X/A, SD/−Ade/−His/−Leu/−Trp/X-α-Gal/AbA.

The Y2H system was used to identify host cellular proteins that interact with the IAV M2 protein. As bait, we used full-length M2 and the M2 CT domain of two different virus strains: A/Sichuan/1/2009 (SC09) (H1N1) and A/Anhui/2/05 (AH05) (H5N1). A cDNA library prepared from a mixed human cell culture (A549, HEK293T, THP-1, and U251) was used as prey. More than 8 × 10^8^ yeast colonies were screened from the library, which contained >2 × 10^7^ independent clones, representing a >40-fold screening of the library. Putative positive colonies were selected for growth by using synthetically defined medium lacking leucine and tryptophan (SD/−Leu/−Trp), also known as double dropout (DDO) medium, and plasmids were isolated from positive colonies and sequenced. The in-frame proteins were retransformed into strains carrying the M2 and M2 CT fusion proteins to confirm the interaction with the M2 protein. One specific protein binding partner for M2, TRAPPC6A, was selected for further study due to its abundance in the screen and its specific interaction with both M2 and the M2 CT of the two different virus subtypes (Fig. 1). Full-length TRAPPC6A is a 173-amino-acid protein. The plasmid that was recovered from the Y2H screen containing TRAPPC6A was found to contain the full-length form (NCBI RefSeq accession number NM_024108.2). Its role in modulating virus infection has never been reported.

### M2 interacts with TRAPPC6A and TRAPPC6AΔ in human cells.

**FIG 2:**
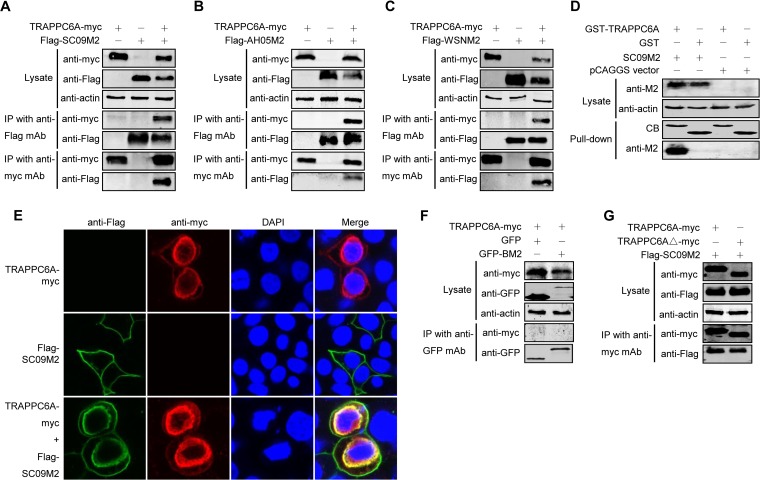
M2 interacts with TRAPPC6A and TRAPPC6AΔ in mammalian cells. (A to C) Plasmids expressing TRAPPC6A-Myc and Flag-SC09M2 (A), TRAPPC6A-Myc and Flag-AH05M2 (B), or TRAPPC6A-Myc and Flag-WSNM2 (C) were transfected individually or in combination, as indicated, in HEK293T cells. Forty-eight hours after transfection, cell lysates were immunoprecipitated with a mouse anti-Flag MAb or a mouse anti-Myc MAb and were subjected to Western blotting with a rabbit anti-Flag polyclonal antibody or a rabbit anti-Myc polyclonal antibody to reveal the presence of M2 and TRAPPC6A, respectively. (D) Western blotting of proteins bound to GST alone or to GST-TRAPPC6A. HEK293T cells transfected with pCAGGS-SC09M2 or with the pCAGGS vector were lysed with IP buffer, and the lysate was incubated with purified GST or GST-TRAPPC6A and then subjected to a pulldown assay. Equal volumes of proteins bound to the beads and the original cell lysates (5% of the input) were examined by Western blotting using a mouse anti-M2 MAb or a mouse anti-actin MAb, respectively. The GST-tagged proteins in the eluates were detected by Coomassie blue (CB) staining. (E) Confocal analysis of the distribution of M2 and TRAPPC6A proteins in A549 cells. pCAGGS-TRAPPC6A-myc and pCAGGS-Flag-SC09M2 were transfected individually or in combination into A549 cells and assessed by immunofluorescence staining. IAV M2 was detected with a mouse anti-Flag MAb and visualized with Alexa Fluor 488 (green). TRAPPC6A was detected with a rabbit anti-Myc polyclonal antibody and visualized with Alexa Fluor 546 (red). Yellow indicates colocalization of Alexa Fluor 546 and 488 in the merged image. (F) pCAGGS-TRAPPC6A-myc was cotransfected with pEGFP-C1 or pEGFP-C1-BM2 into HEK293T cells for 48 h before the cells were lysed. Following immunoprecipitation of the cell lysates with a mouse anti-GFP MAb, the immunoprecipitates were analyzed by Western blotting using a rabbit anti-GFP polyclonal antibody or a rabbit anti-Myc polyclonal antibody to reveal the presence of BM2 and TRAPPC6A, respectively. (G) Co-IP of M2 and TRAPPC6AΔ. pCAGGS-Flag-SC09M2 was cotransfected with pCAGGS-TRAPPC6A-myc or pCAGGS-TRAPPC6AΔ-myc into HEK293T cells. Forty-eight hours after transfection, cell lysates were immunoprecipitated with a mouse anti-Myc MAb and subjected to Western blotting with a rabbit anti-Flag polyclonal antibody or a rabbit anti-Myc polyclonal antibody to reveal the presence of M2 and TRAPPC6A or TRAPPC6AΔ, respectively.

To determine whether M2 interacts with TRAPPC6A in human cells, we performed co-IP experiments using primary antibodies that recognize both proteins. HEK293T cells were transfected with either Flag-tagged SC09 M2, Myc-tagged TRAPPC6A, or both. Cell lysates were collected from transfected cells and immunoprecipitated with an anti-Flag monoclonal antibody (MAb), followed by Western blotting with a rabbit anti-Flag antibody for the detection of M2 and a rabbit anti-Myc antibody to reveal the presence of TRAPPC6A. TRAPPC6A-Myc was readily coimmunoprecipitated with Flag-SC09 M2 (Fig. 2A), demonstrating their physical interaction *in vivo*. The specificity of the M2-TRAPPC6A interaction was further demonstrated by the detection of Flag-SC09 M2 in a reverse co-IP experiment using an anti-Myc MAb and by the absence of Flag-SC09 M2 on the blot from cells transfected with Flag-SC09 M2 alone (Fig. 2A). The physical interaction between TRAPPC6A and M2 was also confirmed with two other influenza viruses, AH05 and A/WSN/33 (WSN) (Fig. 2B and C), suggesting that the interaction between TRAPPC6A and M2 is a property common to influenza A viruses. To further determine whether the interaction between TRAPPC6A and M2 can occur *in vitro*, we performed a glutathione *S*-transferase (GST) pulldown assay by using purified GST-TRAPPC6A or GST alone incubated with HEK293T lysates containing exogenously expressed SC09 M2 protein. The results showed that SC09 M2 was specifically pulled down with GST-TRAPPC6A but not GST alone (Fig. 2D), indicating that TRAPPC6A can interact with influenza virus M2 *in vitro*.

The localization of M2 and TRAPPC6A was examined by using immunofluorescence and confocal microscopy in A549 cells transfected with Flag-SC09 M2 and TRAPPC6A-Myc individually or in combination (Fig. 2E). M2 was predominantly distributed in the plasma membrane, and TRAPPC6A was mainly distributed in the perinuclear region, with less protein in the cytoplasm and plasma membrane when they were transfected individually into cells. When they were cotransfected, the localization of TRAPPC6A was unchanged. However, the localization of M2 was significantly different than when it was transfected alone. Notably, M2 and TRAPPC6A clearly colocalized in the perinuclear region upon coexpression.

To determine whether the interaction between TRAPPC6A and M2 is specific to influenza A virus or can be extended to influenza B virus, we performed an additional co-IP experiment with TRAPPC6A and BM2 of the B/Jilin/20/2003 virus. We found that TRAPPC6A was not coimmunoprecipitated with BM2 fused to green fluorescent protein (GFP) (Fig. 2F), thus demonstrating the specificity of the interaction between TRAPPC6A and M2 of influenza A virus.

TRAPPC6AΔ is identical to full-length TRAPPC6A except for an N-terminal internal deletion of 14 amino acids. We therefore examined whether the specificity of the interaction between full-length TRAPPC6A and M2 extended to TRAPPC6AΔ. We performed a co-IP assay with HEK293T cells transfected with Flag-tagged SC09 M2 and Myc-tagged TRAPPC6A or TRAPPC6AΔ. The cell lysates were incubated with an anti-Myc MAb, followed by Western blotting to detect the coimmunoprecipitated proteins. Flag-tagged M2 was coimmunoprecipitated with both Myc-tagged TRAPPC6A and TRAPPC6AΔ (Fig. 2G). These results indicate that both TRAPPC6A and TRAPPC6AΔ interact with M2.

### The leucine residue at position 96 of M2 mediates its interaction with TRAPPC6A/TRAPPC6AΔ.

**FIG 3:**
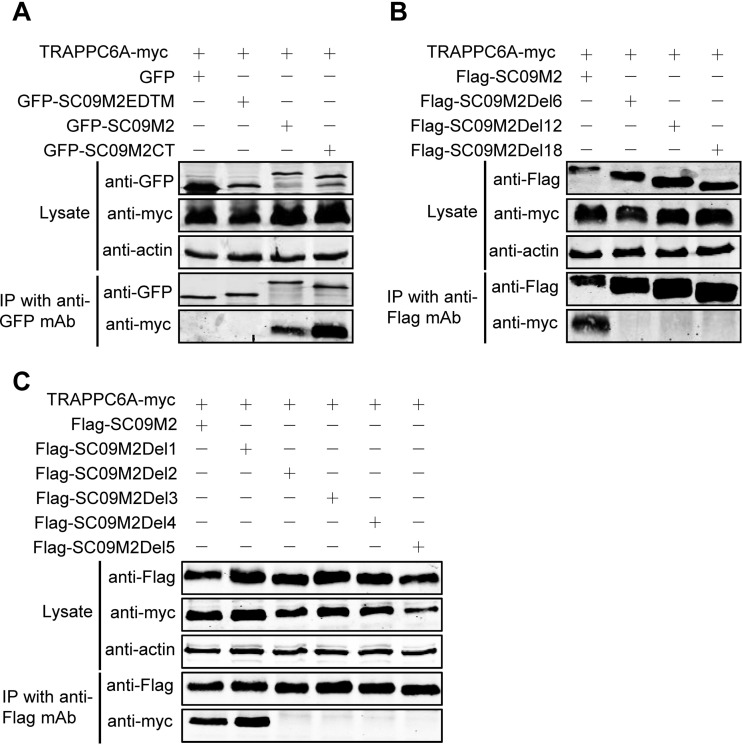
A leucine residue at position 96 of M2 is required for the TRAPPC6A interaction. (A) pCAGGS-TRAPPC6A-myc was cotransfected with pEGFP-C1, pEGFP-C1-SC09 M2, pEGFP-C1-SC09 M2EDTM, or pEGFP-C1-SC09 M2CT into HEK293T cells for 48 h before preparation for cell lysates. Following immunoprecipitation with a mouse anti-GFP MAb, the immunoprecipitates were analyzed by Western blotting using a rabbit anti-GFP polyclonal antibody and a rabbit anti-Myc polyclonal antibody to reveal the presence of M2 and TRAPPC6A, respectively. (B and C) Plasmids expressing TRAPPC6A-myc and Flag-SC09M2 or Flag-SC09M2 with different amino acid deletions in the C terminus were cotransfected into HEK293T cells for 48 h before the preparation of cell lysates. Following immunoprecipitation with a mouse anti-Flag MAb, the immunoprecipitates were analyzed by Western blotting using a rabbit anti-Flag polyclonal antibody or a rabbit anti-Myc polyclonal antibody to reveal the presence of M2 and TRAPPC6A, respectively.

To define the specific binding domain and residue for TRAPPC6A/TRAPPC6AΔ in M2, we first validated the finding of the Y2H experiment that the M2 CT mediates the interaction with TRAPPC6A in human cells. SC09 M2 was divided into two fragments according to its function, the CT fragment and the EDTM fragment, which includes the ectodomain and the transmembrane domain. Full-length M2 and the two fragments were fused to GFP in the C terminus, resulting in the construction of pEGFP-C1-SC09 M2, pEGFP-C1-SC09 M2EDTM, and pEGFP-C1-SC09 M2CT. We then coexpressed these fusion constructs with Myc-tagged TRAPPC6A in HEK293T cells and performed a co-IP experiment with a mouse anti-GFP MAb, followed by Western blotting with a rabbit anti-GFP polyclonal antibody for the detection of GFP-fused SC09 M2 and a rabbit anti-Myc antibody for the detection of TRAPPC6A. We found that both full-length SC09 M2 and the SC09 M2 CT formed a complex with TRAPPC6A but that the SC09 M2 EDTM fragment failed to associate with TRAPPC6A (Fig. 3A), indicating that the M2 CT domain is critical for the binding of M2 with TRAPPC6A. Next, we attempted to narrow down the regions in the M2 CT that are involved in the interaction with TRAPPC6A. Three M2 truncation mutants were constructed with 6-, 12-, and 18-amino-acid deletions from the C terminus, respectively, and used in co-IP assays. As shown in Fig. 3B, full-length SC09 M2 interacted with TRAPPC6A, whereas SC09 M2Del6, SC09 M2Del12, and SC09 M2Del18 were not coimmunoprecipitated with TRAPPC6A, indicating that the 6 amino acids in the C terminus of M2 are important for the interaction with TRAPPC6A. To determine the specific residue among these 6 amino acids responsible for the binding of M2 to TRAPPC6A, we performed further co-IP experiments with C-terminally truncated SC09 M2 constructs of 1 amino acid deletion at a time. Both full-length SC09 M2 and SC09 M2Del1, which lacks 1 amino acid from the C terminus, interacted with TRAPPC6A, whereas SC09 M2Del2, SC09 M2Del3, SC09 M2Del4, and SC09 M2Del5, which lack 2, 3, 4, and 5 amino acids from the C terminus, respectively, could not bind to the TRAPPC6A protein (Fig. 3C). Similarly, we demonstrated that SC09 M2Del1 interacted with TRAPPC6AΔ in the co-IP assay, whereas SC09 M2Del2 did not (data not shown). IAV M2 is 97 amino acids long. These data indicate that the leucine residue at position 96 of SC09 M2 is critical for the interaction of M2 with TRAPPC6A/TRAPPC6AΔ.

### Mutation at position 96 of M2 affects its interaction with TRAPPC6A.

**FIG 4:**
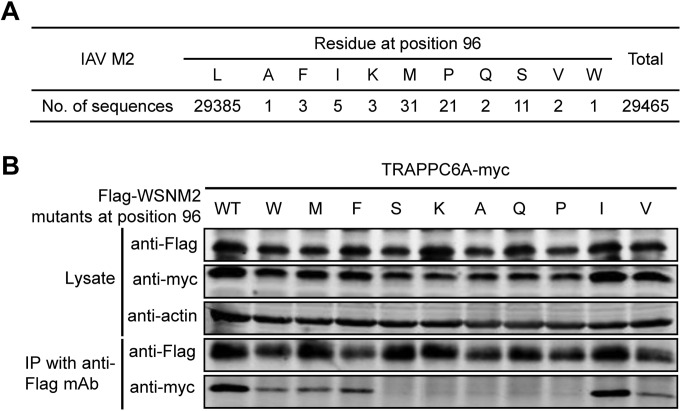
Mutation at position 96 of M2 affects its interaction with TRAPPC6A. (A) Sequence analysis of IAV M2 at position 96. All of the IAV M2 sequences deposited in GenBank by 6 July 2014 were downloaded. The identity of the amino acids at position 96 was statistically analyzed. (B) Plasmids expressing TRAPPC6A-Myc and Flag-WSNM2 or Flag-WSNM2 with different mutations at position 96 were cotransfected into HEK293T cells for 48 h before the preparation of cell lysates. Following immunoprecipitation with a mouse anti-Flag MAb, the immunoprecipitates were analyzed by Western blotting using a rabbit anti-Flag polyclonal antibody or a rabbit anti-Myc polyclonal antibody to reveal the presence of M2 and TRAPPC6A, respectively.

We demonstrated that the residue at position 96 of M2 is critical for its interaction with TRAPPC6A/TRAPPC6AΔ. To further assess the importance of this residue for the interaction with TRAPPC6A/TRAPPC6AΔ, we genetically analyzed all of the influenza virus M2 sequences that were deposited in GenBank by 6 July 2014. Eleven different amino acids were found at position 96 among the 29,465 M2 protein sequences analyzed (Fig. 4A). Strikingly, leucine was highly conserved at this position, accounting for >99.7% of all of the M2 sequences. We then conducted co-IP assays with a series of plasmids encoding WSN M2 with point mutations at position 96; note that wild-type (wt) WSN M2 contains the predominant leucine residue at this position. Substitution of leucine for isoleucine slightly reduced the interaction between M2 and TRAPPC6A (Fig. 4B); substitution of leucine for phenylalanine further reduced the interaction, and substitution of leucine for tryptophan, methionine, or valine resulted in weak interactions, whereas substitution of leucine for alanine, glutamine, proline, serine, or lysine almost abolished the interaction between M2 and TRAPPC6A. These results further demonstrate that the highly conserved 96L residue in M2 is virtually indispensable for the interaction between M2 and TRAPPC6A, suggesting an adaptation of the M2 protein to support its interaction with the host factor TRAPPC6A/TRAPPC6AΔ during IAV evolution.

### TRAPPC6AΔ positively modulates influenza virus infection.

**FIG 5:**
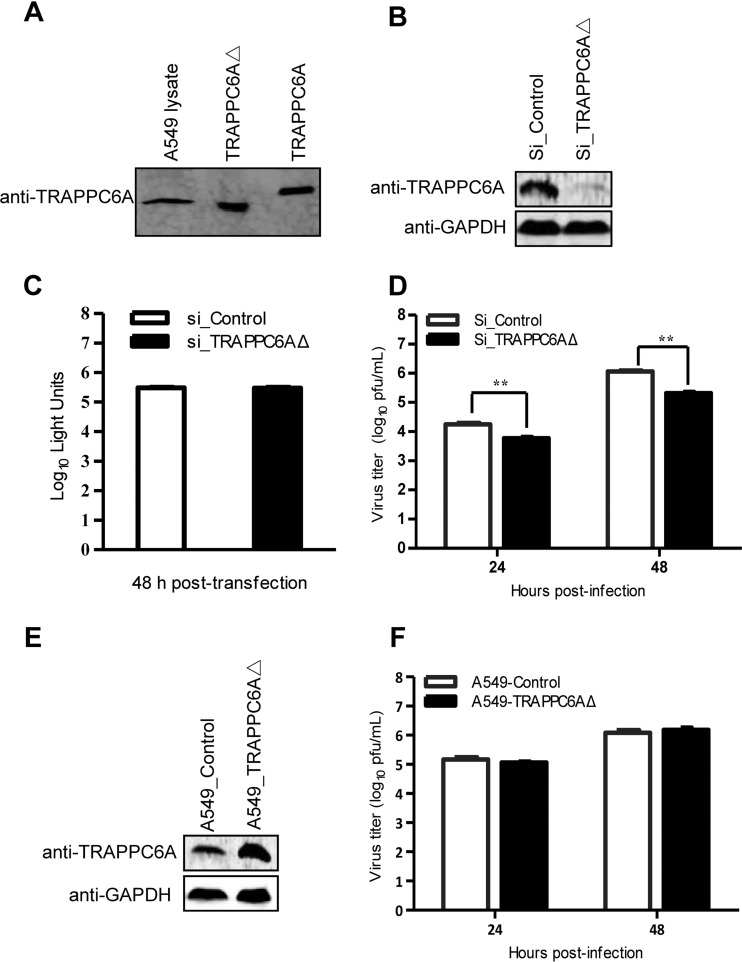
TRAPPC6AΔ positively modulates influenza virus infection. (A) Endogenous expression of TRAPPC6AΔ in A549 cells. Whole lysates of A549 cells grown in 12-well plates were subjected to Western blotting with a rabbit anti-TRAPPC6A polyclonal antibody. HEK293T cell lysates transiently transfected with pCAGGS-TRAPPC6A or pCAGGS-TRAPPC6AΔ were used as a control. (B) siRNA knockdown of TRAPPC6AΔ in A549 cells. A549 cells were transfected with siRNA targeting TRAPPC6AΔ or nontargeting siRNA for 48 h. Whole-cell lysates were then collected and analyzed by Western blotting with a rabbit anti-TRAPPC6A polyclonal antibody. (C) Cell viability of siRNA-treated A549 cells measured by using the CellTiter-Glo assay. A549 cells were transfected with siRNA as described above for panel B. The data are presented as means ± standard deviations for triplicate transfections. (D) Virus replication in siRNA-treated A549 cells. Cells transfected with siRNA as described above for panel B were infected with WSN virus. At 24 and 48 h p.i., supernatants were collected and titrated for infectious virus by plaque assays in MDCK cells. Three independent experiments were performed, and data are shown as means ± standard deviations for triplicates from a representative experiment. **, *P* < 0.01. (E) Stable A549 cell line overexpressing TRAPPC6AΔ. A549 cells were used to establish a stable cell line overexpressing TRAPPC6AΔ by using a retroviral vector. The stable overexpression of TRAPPC6AΔ was confirmed by Western blotting with a rabbit anti-TRAPPC6A polyclonal antibody in comparison with the A549 control cell line transduced with an empty retrovirus. (F) Virus replication in TRAPPC6AΔ-overexpressing A549 cells. WSN virus was used to infect the TRAPPC6AΔ-overexpressing A549 cell line or the A549 control cell line transduced with the empty retrovirus at an MOI of 0.01. Supernatants were collected at 24 and 48 h p.i., and virus titers were determined by plaque assays on MDCK cells. Three independent experiments were performed, and data are shown as means ± standard deviations for triplicates from a representative experiment.

We demonstrated that both TRAPPC6A and TRAPPC6AΔ interact with M2 of IAV. To assess the role of this interaction in influenza virus replication in A549 cells, we first examined the endogenous expression of TRAPPC6A and TRAPPC6AΔ by Western blotting. We found that the rabbit anti-TRAPPC6A polyclonal antibody that we generated recognizes both TRAPPC6A and TRAPPC6AΔ. Full-length TRAPPC6A was not detected in the lysates of A549 cells; however, TRAPPC6AΔ was abundantly expressed (Fig. 5A). We therefore attempted to determine the role of endogenous TRAPPC6AΔ in influenza virus replication in A549 cells by using small interfering RNA (siRNA) targeting. Western blot analyses confirmed that the specific siRNA duplex efficiently reduced cellular TRAPPC6AΔ expression levels and that nontargeting control siRNA did not (Fig. 5B). Furthermore, A549 cells treated with siRNA targeting TRAPPC6AΔ exhibited no difference in viability compared with cells treated with a nontargeting siRNA control at 48 h posttransfection (Fig. 5C). Next, we infected siRNA-transfected cells with influenza virus WSN at a multiplicity of infection (MOI) of 0.01. The cell supernatant was harvested at 24 and 48 h postinfection (p.i.). We found that silencing of TRAPPC6AΔ expression led to a significant decrease in progeny virus titers in cell culture supernatants compared with those of control siRNA-treated cells (Fig. 5D). This result shows that knockdown of cellular TRAPPC6AΔ has an inhibitory effect on the growth of influenza virus.

We then assessed the effect of the overexpression of TRAPPC6AΔ on influenza virus replication. We established an A549 cell line overexpressing TRAPPC6AΔ and an A549 control cell line transduced with an empty retrovirus (Fig. 5E) and infected these cells with the WSN virus at an MOI of 0.01. The virus titers in the supernatant were determined at 24 and 48 h p.i. As shown in Fig. 5F, the overexpression of TRAPPC6AΔ had no observable effect on the replication of influenza virus.

Collectively, these results demonstrate that endogenous TRAPPC6AΔ is essential for efficient influenza virus replication, although its overexpression provides no added benefit for virus growth.

### Influenza virus with M2 that fails to interact with TRAPPC6A/TRAPPC6AΔ is attenuated *in vitro* and *in vivo*.

**FIG 6:**
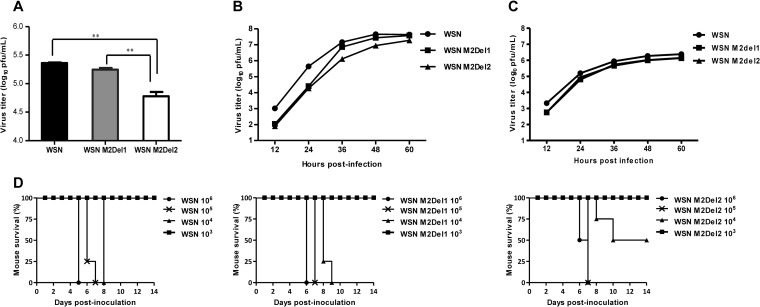
Loss of the interaction between M2 and TRAPPC6A/TRAPPC6AΔ attenuates influenza virus *in vitro* and *in vivo*. (A) Generation of WSN viruses with M2 deletions that prevent the interaction with TRAPPC6A/TRAPPC6AΔ by using reverse genetics. HEK293T cells were transfected with plasmids for the synthesis of eight vRNAs, among which the M vRNA segment encoded wt M2 or M2 mutants lacking 1 or 2 amino acids from the C terminus, together with the four supporting protein expression plasmids. The transfection supernatants were harvested at 48 h posttransfection and titrated by plaque assays in MDCK cells. Three independent experiments were performed, and data are shown as means ± standard deviations for triplicates from a representative experiment. **, *P* < 0.01. (B) Growth kinetics of M2 deletion mutant viruses in A549 cells. A549 cells were infected with the indicated viruses at an MOI of 0.01, and the supernatants were collected at various times and titrated by plaque assays in MDCK cells. Three independent experiments were performed, and the data shown are from a representative experiment. (C) Growth kinetics of M2 deletion mutant viruses in TRAPPC6AΔ-knocked-down A549 cells. A549 cells treated with siRNA targeting TRAPPC6AΔ for 48 h were infected with the indicated viruses at an MOI of 0.01, and the supernatants were collected at various times and titrated by plaque assays in MDCK cells. Three independent experiments were performed; the data shown are from a representative experiment. (D) Pathogenesis of M2 deletion mutant viruses *in vivo*. Mice were inoculated intranasally with the indicated viruses, and mortality was observed for 14 days after inoculation.

M2 lacking 2 residues from the C terminus completely lost the ability to interact with TRAPPC6A/TRAPPC6AΔ. To determine whether this defect in the interaction with TRAPPC6A/TRAPPC6AΔ affects virus replication, we first constructed two pHH21WSN M plasmids that encoded M2 mutants with 1- or 2-amino-acid deletions from the C terminus. HEK293T cells were transfected with plasmids for the synthesis of eight viral RNAs (vRNAs) (the plasmids for the M vRNA segment encode wt M2 or M2 mutants lacking 1 or 2 amino acids from the C terminus), together with the four supporting protein expression plasmids. The transfection supernatants were harvested at 48 h posttransfection and titrated by a plaque assay in MDCK cells. As shown in Fig. 6A, the virus titers in the HEK293T transfection supernatant were 1.8 × 10^5^ PFU/ml for WSN M2Del1 and 2.3 × 10^5^ PFU/ml for the wt virus. In contrast, the virus titer for WSN M2Del2 was 6.0 × 10^4^ PFU/ml, which was considerably lower than those for both WSN M2Del1 and the wt virus. Next, we analyzed the replication kinetics of these three viruses. A549 cells were infected with wt WSN or one of the two mutants at an MOI of 0.01, the supernatants were collected at various times, and their virus titers were assessed (Fig. 6B). The WSN M2Del2 virus showed reduced titers at all time points compared with the wt virus, and the virus titers of WSN M2Del2 were also lower than those of the WSN M2Del1 virus at 36 h to 60 h p.i. We further evaluated the growth of wt WSN, WSN Del1, and WSN Del2 in TRAPPC6AΔ-knocked-down A549 cells. A549 cells treated for 48 h with siRNA targeting TRAPPC6AΔ were infected with wt WSN, WSN Del1, or WSN Del2 at an MOI of 0.01, and supernatants were collected at different time points and titrated in MDCK cells. Of note, the growth of WSN M2Del2 was the same as that of WSN M2Del1 and was also similar to that of wt WSN in TRAPPC6AΔ-knocked-down A549 cells at all time points (Fig. 6C). These data further demonstrate that the interaction between M2 and TRAPPC6AΔ is essential for the optimal replication of influenza virus.

To determine whether the M2-TRAPPC6A/TRAPPC6AΔ interaction has any effect on the virulence of influenza virus *in vivo*, female BALB/c mice were intranasally infected with the WSN, WSN M2Del1, or WSN M2Del2 virus. The mice were observed for 14 days after inoculation, and their body weight and survival status were checked daily. When mice were infected with a low dose (i.e., 10^3^ PFU per animal) of these viruses, all of the mice survived (Fig. 6D). When mice were infected with high doses (i.e., 10^5^ and 10^6^ PFU per animal) of the viruses, all of the mice died. However, half of the mice survived when they were infected with 10^4^ PFU of WSN M2Del2. In contrast, all of the mice died when they were infected with 10^4^ PFU of either wt WSN or WSN M2Del1. These results indicate that the loss of the M2-TRAPPC6A/TRAPPC6AΔ interaction leads to a modest attenuation of influenza virus virulence *in vivo*.

### Modulation of TRAPPC6AΔ expression alters the cell surface expression of the M2 protein.

**FIG 7:**
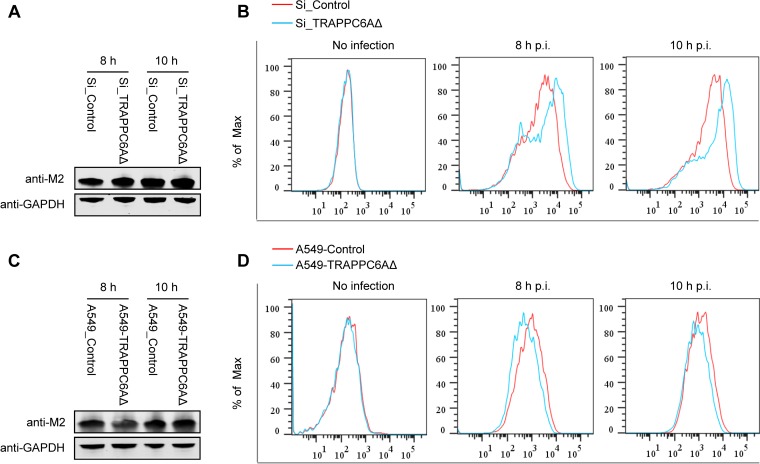

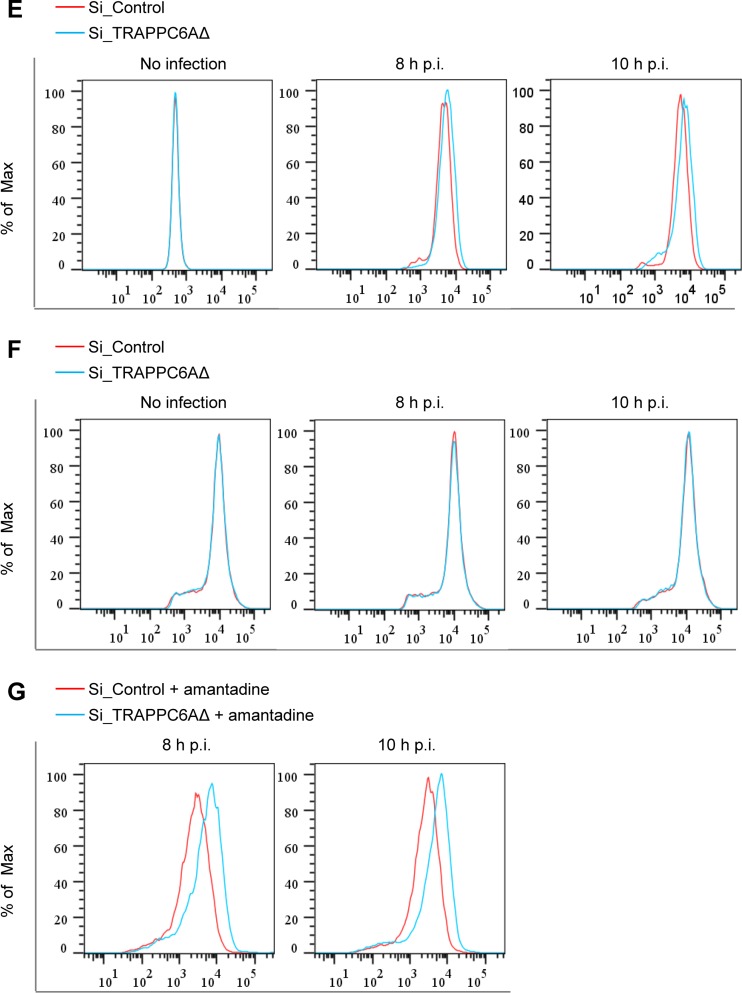
Effect of modulation of TRAPPC6AΔ expression on the cell surface expression of viral and cellular proteins. (A) A549 cells were transfected with siRNA targeting TRAPPC6AΔ or with nontargeting siRNA for 48 h and were then infected with the WSN virus at an MOI of 3. Cell lysates were processed at 8 and 10 h p.i. and subjected to Western blotting using a mouse anti-M2 MAb to detect the expression level of M2. (B) A549 cells were treated with siRNA and infected with the WSN virus as described above for panel A. Cells were fixed at 8 and 10 h p.i., left nonpermeabilized, and stained with the mouse anti-M2 MAb and Alexa Fluor 488-conjugated donkey anti-mouse IgG(H+L) for M2 surface expression analysis by flow cytometry. The graph shows the fluorescence intensity of M2 surface expression. (C) The TRAPPC6AΔ-overexpressing A549 cell line or the A549 control cell line transduced with an empty retrovirus was infected with the WSN virus at an MOI of 3. Cell lysates were processed at 8 and 10 h p.i. and subjected to Western blotting using a mouse anti-M2 MAb to detect the expression level of M2. (D) The TRAPPC6AΔ-overexpressing A549 cell line or the A549 control cell line transduced with an empty retrovirus was infected with the WSN virus at an MOI of 3. The cell surface expression of M2 was analyzed by flow cytometry at 8 and 10 h p.i. as described above for panel B. (E) A549 cells were treated with siRNA and infected with the WSN virus as described above for panel A. The cell surface expression of HA was analyzed by flow cytometry at 8 and 10 h p.i. as described above for panel B by using the rabbit anti-HA polyclonal antibody and Alexa Fluor 488-conjugated goat anti-rabbit IgG(H+L). (F) A549 cells were treated with siRNA and infected with the WSN virus as described above for panel A. The cell surface expression of FGF2 was analyzed by flow cytometry at 8 and 10 h p.i. as described above for panel B by using the rabbit anti-FGF2 MAb and Alexa Fluor 488-conjugated goat anti-rabbit IgG(H+L). (G) A549 cells were treated with siRNA and infected with the WSN virus as described above for panel A. At 2 h p.i., the culture medium was replaced with medium supplemented with 25 μM amantadine. The cell surface expression of M2 was analyzed by flow cytometry at 8 and 10 h p.i. as described above for panel B.

Upon expression, the influenza virus M2 protein is transported to the cellular plasma membrane through the protein secretion pathway to participate in virus budding and release. TRAPPC6AΔ, occasionally termed TRAPPC6A in some studies (39, 44), is a subunit of the transport protein particle (TRAPP) complex that is involved in protein vesicle trafficking in mammalian cells (36, 37). Compared with full-length TRAPPC6A, TRAPPC6AΔ is abundantly expressed in A549 cells. We hypothesized that modulation of TRAPPC6AΔ expression may affect the cell surface expression of M2. We therefore quantified the cell surface expression levels of the M2 protein by using flow cytometry on A549 cells that were transfected with an siRNA targeting TRAPPC6AΔ or nontargeting siRNA and subsequently infected with the WSN virus at an MOI of 3. Western blotting showed that the siRNA knockdown of TRAPPC6AΔ did not affect the expression level of M2 in infected cells (Fig. 7A). However, M2 surface expression was significantly different between A549 cells treated with targeting siRNA and those treated with nontargeting siRNA at both 8 h and 10 h p.i. Notably, the intensity of M2 surface expression was greatly increased when the expression of TRAPPC6AΔ was knocked down by targeting siRNA (Fig. 7B), indicating that the expression of TRAPPC6AΔ slowed the transport of M2 to the plasma membrane. To corroborate this finding, we also performed flow cytometry analysis on the TRAPPC6AΔ-overexpressing A549 stable cell line infected with the WSN virus at an MOI of 3. As shown in Fig. 7C, the expression level of M2 was not affected by the overexpression of TRAPPC6AΔ. However, the overexpression of TRAPPC6AΔ resulted in a “left shift” in the graph of M2 surface expression compared with that of the A549 control cell line transduced with an empty retrovirus (Fig. 7D), indicating that the overexpression of TRAPPC6AΔ reduced the surface expression of M2. Overall, these results suggest that TRAPPC6AΔ acts to slow the trafficking of M2 along the secretory pathway.

We next determined whether TRAPPC6AΔ affects the transport of the viral HA protein, which also traffics to the plasma membrane through the Golgi apparatus. siRNA-treated A549 cells were infected with the WSN virus, and the cell surface expression level of the HA protein was determined by flow cytometry using a rabbit anti-HA polyclonal antibody at 8 and 10 h p.i. We found that the cell surface expression level of HA was very slightly increased in TRAPPC6AΔ-knocked-down cells compared with nontargeting siRNA-treated cells at both time points (Fig. 7E). Previous studies have shown that the expression of the influenza virus M2 protein slows the transport of viral HA as well as other integral membrane glycoproteins through the Golgi apparatus (45, 46). Therefore, this minor enhancement of the transport of HA might be an indirect effect of the accelerated cell surface expression of the M2 protein upon the knockdown of TRAPPC6AΔ. We also determined the effect of TRAPPC6AΔ knockdown on the transport of a cellular protein, fibroblast growth factor 2 (FGF2), which is transported to the cell surface through a Golgi-independent secretion pathway (47). siRNA-treated A549 cells were infected with the WSN virus, and the cell surface expression level of the FGF2 protein was determined by flow cytometry using an anti-FGF2 MAb at 8 and 10 h p.i. As shown in Fig. 7F, the cell surface expression levels of FGF2 were similar between nontargeting siRNA- and TRAPPC6AΔ siRNA-treated A549 cells at both time points. These results demonstrate that the modulation of protein transport by TRAPPC6AΔ is not a general phenomenon but rather relies on the interaction between M2 and TRAPPC6AΔ.

The acidic pH of the TGN is equilibrated with the pH of the cytoplasm via the activation of the M2 proton-selective ion channel, which in turn affects the kinetics of the delivery of apical membrane proteins to the cell surface and can be blocked by the addition of the ion channel blocker amantadine (48). To ensure that the observed effect of TRAPP6AΔ on the cell surface expression of M2 is not caused by the ion channel activity of M2, we repeated the M2 cell surface expression experiment with A549 cells that were treated with TRAPPC6AΔ-specific siRNA or nontargeting siRNA and subsequently infected with the WSN virus at an MOI of 3. At 2 h p.i., the cells were treated with 25 μM amantadine. At 8 h and 10 h p.i., M2 surface expression was quantified by using flow cytometry. We found that the knockdown of TRAPP6AΔ expression still increased the cell surface expression of M2 when infected cells were treated with amantadine (Fig. 7G). These results indicate that TRAPPC6AΔ can indeed affect the apical transport of M2.

### Regulation of TRAPPC6AΔ during posttranslational transport of M2 is a dynamic process.

**FIG 8:**
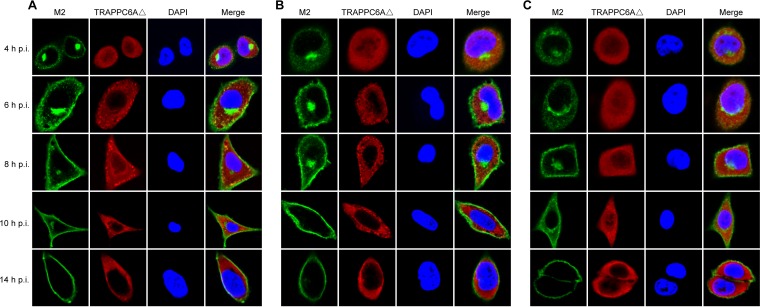
Dynamics of the interaction of M2 and TRAPPC6AΔ in wt and mutant WSN virus-infected cells. A549 cells were infected with wt influenza virus WSN (A), or one of the M2 deletion mutants WSN M2Del1 (B) and WSN M2Del2 (C), at an MOI of 5. At 4, 6, 8, 10, and 14 h p.i., the infected cells were fixed and stained with mouse anti-M2 MAb 14C2 and rabbit anti-TRAPPC6A polyclonal antibody, followed by incubation with Alexa Fluor 488 donkey anti-mouse IgG(H+L) (green) and Alexa Fluor 546 donkey anti-rabbit IgG(H+L) (red). Nuclei were stained with DAPI.

**FIG 9:**
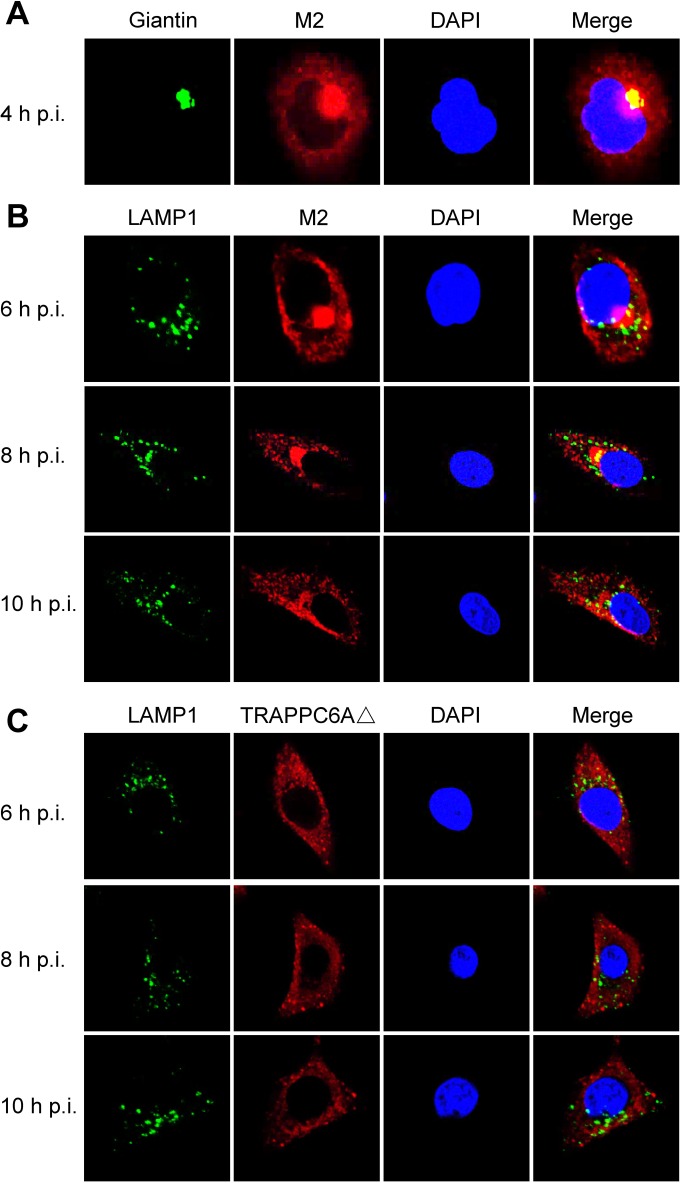
Confocal microscopy of WSN virus-infected cells stained for the Golgi apparatus or lysosomes. A549 cells were infected with the wt WSN virus at an MOI of 5. At the indicated time points, infected cells were fixed and stained with mouse anti-Giantin MAb and rabbit anti-M2 polyclonal antibody (A), mouse anti-LAMP1 MAb and rabbit anti-M2 polyclonal antibody (B), or mouse anti-LAMP1 MAb and rabbit anti-TRAPPC6A polyclonal antibody (C), followed by incubation with Alexa Fluor 488 donkey anti-mouse IgG(H+L) (green) and Alexa Fluor 546 donkey anti-rabbit IgG(H+L) (red). Nuclei were stained with DAPI.

We demonstrated that endogenous TRAPPC6AΔ slows the trafficking of M2 to the cell surface, which is beneficial for virus replication. We next attempted to further determine the dynamic changes in TRAPPC6AΔ during the transport of M2 to the plasma membrane. We performed a time course confocal experiment to examine the M2-TRAPPC6AΔ interaction during virus replication. A549 cells were infected with wt WSN, WSN M2Del1, or WSN M2Del2 at an MOI of 5. At 4 h, 6 h, 8 h, 10 h, and 14 h p.i., the localization of M2 and TRAPPC6AΔ was determined. In noninfected control cells, endogenous TRAPPC6AΔ was evenly distributed in the cytoplasm (data not shown). In WSN-infected cells, M2 clearly accumulated in the area of the Golgi apparatus that stained positive for Giantin and was also dispersed in the cytoplasm at 4 h p.i. (Fig. 8A and 9A). This distribution pattern is consistent with data from previous reports on the distribution of influenza virus M2 (40). TRAPPC6AΔ was diffusely distributed throughout the cytoplasm at 4 h p.i. (Fig. 8A), a pattern that was also observed in noninfected control cells. At 6 h, 8 h, and 10 h, M2 exhibited many punctate areas or dots in the cytoplasm, indicating that M2 was abundantly incorporated into apical vesicles and released from the Golgi apparatus. Importantly, TRAPPC6AΔ also changed its diffusion state and accumulated in the cytoplasm as punctate areas or dots. As expected, M2 and TRAPPC6AΔ were colocalized at the punctate dots, indicating that TRAPPC6AΔ was exerting its role in regulating the pace of M2 trafficking to the plasma membrane. At 14 h p.i., most of the M2 protein was distributed at the plasma membrane, suggesting that the secretory transport process was largely completed. Notably, TRAPPC6AΔ was no longer in an accumulated state but had returned to the same diffuse state as that in noninfected cells, a sign that TRAPPC6AΔ had completed its task in the M2 transport process at this time point. When cells were stained for the lysosome marker LAMP1 during infection, the partial colocalization of M2 with LAMP1 was seen only when M2 accumulated densely in the Golgi region, and TRAPPC6AΔ did not colocalize with LAMP1 at any time point (Fig. 9B and C), indicating that the role of TRAPPC6AΔ is not to target M2 to the lysosome during its posttranslational transport to the apical cell surface. Next, we found that TRAPPC6AΔ also plays a role in the transport of M2 in WSN M2Del1-infected A549 cells. In these cells, the distribution of M2 and TRAPPC6AΔ was largely the same as that in wt WSN-infected cells (Fig. 8B). TRAPPC6AΔ was colocalized with M2 in punctate dots at 6 h, 8 h, and 10 h p.i., whereas it was diffuse in the cytoplasm at early and late time points (4 h and 14 h p.i.). Finally, we examined the distribution of M2 and TRAPPC6AΔ in WSN M2Del2-infected cells (Fig. 8C). The distribution of M2 was similar to that in WSN- and WSN M2Del1-infected cells at 4 h and 14 h p.i., whereas M2 formed fewer punctate dots in the cytoplasm at 6 h, 8 h, and 10 h p.i. Of note, TRAPPC6AΔ did not form punctate dots and was diffuse in the cytoplasm across all time points, as was observed in noninfected control cells. No colocalization between M2 and TRAPPC6AΔ was observed in WSN M2Del2-infected cells, which is consistent with the finding that TRAPPC6AΔ does not interact with M2 that lacks 2 residues at positions 96 and 97. Taken together, these results show the changes in the distribution of TRAPPC6AΔ in a dynamic process during influenza virus infection and demonstrate that TRAPPC6AΔ accumulates in punctate dots, where it may exert its role to slow M2 trafficking to the virus budding site on the plasma membrane and thus modulate virus propagation.

## DISCUSSION

The influenza virus life cycle is finely regulated in order to maintain optimal virus fitness, infectivity, and transmissibility. The viral M2 protein, which is synthesized during the late stage of the virus life cycle (42), is transported to the plasma membrane and assembled into virus particles as one of the envelope proteins. Once synthesized, M2 is translocated to the endoplasmic reticulum (ER), travels to the Golgi apparatus, and is sorted at the TGN for transport to the plasma membrane. This is the general principle for the transport of secreted proteins in cells. However, the details of influenza virus M2 protein trafficking and the molecules with which it interacts during this process are as yet largely unknown. In this study, we identified a cellular protein, TRAPPC6A, as a novel interacting partner of the influenza virus M2 protein by using Y2H screening. We demonstrated that both TRAPPC6A and its N-terminal internal-deletion isoform, TRAPPC6AΔ, can interact with M2. We found that 96L of M2 is the key residue in mediating the interaction with TRAPPC6A/TRAPPC6AΔ and that this residue is highly conserved in M2 proteins across IAVs, which suggests that this might be the outcome of the adaptation of the viral M2 protein to the host cellular protein TRAPPC6A/TRAPPC6AΔ during virus evolution. Our results indicate that TRAPPC6AΔ acts to slow the trafficking of M2 to the plasma membrane and positively modulates the production of influenza virus progeny.

We demonstrated that TRAPPC6AΔ is involved in the posttranslational transport of influenza virus M2 to the plasma membrane. Flow cytometry analyses showed that siRNA knockdown of TRAPPC6AΔ increased the surface expression of M2, whereas stable overexpression of TRAPPC6AΔ reduced its surface expression. We also visualized dynamic changes in TRAPPC6AΔ during the trafficking of M2 to the plasma membrane. In WSN- and WSN M2Del1-infected cells, TRAPPC6AΔ accumulated in punctate dots, possibly transport vesicles, where it is colocalized with M2. Once the transport of M2 to the plasma membrane is complete, TRAPPC6AΔ returns to its diffuse state. Strikingly, TRAPPC6AΔ maintained its diffuse state throughout the whole infection process, and no colocalization between M2 and TRAPPC6AΔ was observed in WSN M2Del2-infected cells. Based on these data, we conclude that the physical interaction between TRAPPC6AΔ and M2 slows M2 to go through the secretory pathway and arrive at the plasma membrane. However, whether TRAPPC6AΔ alone is sufficient for this activity or whether it has to rely on the function of TRAPP as a complex to modulate M2 transport remains to be investigated.

Our data demonstrated that TRAPPC6AΔ is required for the efficient growth of influenza virus. Upon the knockdown of TRAPPC6AΔ with siRNA in A549 cells, the replication of WSN virus was significantly reduced. However, the stable overexpression of TRAPPC6AΔ in A549 cells did not result in increased virus replication. These results suggest that the endogenous expression of TRAPPC6AΔ might be enough to fulfill its function in regulating M2 transport, and therefore, the overexpression of TRAPPC6AΔ provided no additional benefit. Another possibility is that the overexpression of TRAPPC6AΔ may overwhelm or alter the transport machinery of the host cells. In this case, there would also be no further promotion of virus growth when TRAPPC6AΔ was overexpressed. In fact, it has been reported that the overexpression of subunits of the TRAPP complex can disrupt the architecture of microtubules in such a way that the astral appearance becomes disorganized, frequently presenting as a circular array (49). In this study, when TRAPPC6AΔ was transiently overexpressed in A549 cells, we observed that most of the TRAPPC6AΔ accumulated in the perinuclear region. This distribution pattern differs from the diffuse pattern of endogenous TRAPPC6AΔ in A549 cells, indicating that the overexpression of TRAPPC6AΔ may have altered the transport machinery in some way.

Previous studies have shown that the M2 protein can slow the trafficking of the influenza virus HA protein and other integral membrane glycoproteins through the Golgi complex (45). In addition, the ion-channel-active form of M2 reaches the plasma membrane more slowly than does the inactive form (48). Thus, the ion channel activity of M2 not only may regulate the trafficking of other proteins along the secretory pathway but also may affect the transport of M2 *per se* to the plasma membrane. Here, we identified TRAPPC6AΔ as a cellular factor that regulates the trafficking of M2 to the plasma membrane. These are the first data to demonstrate that the quantity and timing of M2 transport to the plasma membrane are also controlled by interacting host factors in addition to the M2 ion channel activity. Our results also indicate that the transport of the M2 protein to the cell surface does not follow the “more is better” concept. In fact, the ratio of M2 molecules to HA molecules is only in the range of 1:10 to 1:100 on influenza virus particles (50).

As a viral pathogen, influenza virus has to utilize the host cellular machinery for many aspects of its life cycle. As a result, the virus must adapt to host factors during the virus life cycle. One type of adaptation occurs when the virus infects a new host. For instance, avian influenza viruses are prone to acquiring the adaptive PB2 mutation E627K or D701N when they cross the species barrier to infect humans or other mammals (51–56). This type of adaptation might be driven by host-specific cellular factors, as evidenced by the recent identification of ANP32A in driving the PB2 E627K mutation in humans (57). The other type of adaptation is conserved in all influenza viruses regardless of their host species origin. The conservation of such adaptations in viral proteins might be required to maintain protein structure stability or constrained by certain host factors to achieve virus fitness. In this study, we found that the 96L residue is highly conserved in the M2 proteins of influenza viruses isolated from different host species. When the conserved leucine was mutagenized to other residues, the interaction between M2 and TRAPPC6A was diminished or even disappeared. Our data strongly imply that the high level of conservation of 96L is driven by its interacting host factor, TRAPPC6A/TRAPPC6AΔ. Although we assessed the interaction between M2 and TRAPPC6A/TRAPPC6AΔ only in human cells, it is reasonable to assume that such an interaction may also occur in other host species.

M2 is the smallest structural protein of influenza virus but plays multiple roles in the virus life cycle. Although small, M2 can bind to host factors to fulfill its function or can be the target of host restriction factors. So far, several interacting partners of M2 have been reported (40–43). Two of these studies mapped the functional domain in M2 that physically interacts with the binding partners. In one study, the CT domain of M2 was shown to interact directly with the essential autophagy protein LC3 and to promote LC3 relocalization to the unexpected destination of the plasma membrane, thus inhibiting autophagy and enhancing virion stability (41). In another study, Ma et al. reported that AnxA6 interacts with the CT domain of M2 and impairs the budding and release of progeny viruses (40). Here, we demonstrated that TRAPPC6AΔ interacts with the cytoplasmic tail of M2 and slows its transport to the plasma membrane. Together, those studies and ours point out that the CT domain of M2 is a hot region in mediating interactions with host cellular factors. Influenza virus M2 has the longest cytoplasmic tail among the three viral transmembrane proteins (24). As research continues, more partners that interact with the M2 cytoplasmic tail are expected to be discovered, along with more details about the functions of M2.

In conclusion, we identified TRAPPC6A and its N-terminal internal deletion isoform, TRAPPC6AΔ, as novel host factors that interact with influenza virus M2 and revealed that TRAPPC6AΔ is involved in the posttranslational transport of influenza virus M2 and positively modulates virus propagation. This is the first study to identify a role for TRAPP complex subunits in the transport of viral proteins. The identification of a single leucine residue at position 96 of M2 in mediating the interaction between M2 and TRAPPC6A/TRAPPC6AΔ suggests that key residues responsible for the interactions between other viral proteins and host cellular factors may also exist, and their discovery could provide targets for the design of novel therapeutic agents.

## MATERIALS AND METHODS

### Cells and viruses.

Human embryonic kidney cells (HEK293T), human glial cells (U251), human lung carcinoma cells (A549), human monocytic cells (THP-1), and Madin-Darby canine kidney (MDCK) cells were cultured in the following media: Dulbecco's modified Eagle's medium (DMEM; Life Technologies, Grand Island, NY) supplemented with 10% fetal bovine serum (FBS; Sigma-Aldrich, St. Louis, MO) (HEK293T and U251), F-12K medium (Life Technologies) supplemented with 10% FBS (A549), RPMI 1640 medium (Life Technologies) supplemented with 10% FBS (THP-1), and minimum essential medium (MEM; Life Technologies) containing 5% newborn calf serum (NCS; Sigma-Aldrich) (MDCK). All media contained 100 U/ml penicillin and 100 μg/ml streptomycin (Life Technologies). All cells were maintained in a humidified incubator containing 5% CO_2_ at 37°C. The A/WSN/33 (WSN) (H1N1) strain of influenza virus was grown in MDCK cells cultured in MEM supplemented with 0.3% bovine serum albumin (BSA; Sigma-Aldrich) and 0.5 μg/ml l-1-tosylamide-2-phenylmethyl chloromethyl ketone (TPCK)-treated trypsin (Worthington, Lakewood, NJ).

### Yeast two-hybrid assay.

A matchmaker Y2H system (Clontech, Mountain View, CA) was used to screen for host proteins that interact with M2. The bait protein, containing the full-length M2 protein or the M2 CT domain (amino acids 44 to 97) of A/Anhui/2/05 (AH05) (H5N1) or 2009 pandemic H1N1 virus A/Sichuan/1/2009 (SC09), respectively, was expressed with an N-terminal fusion to the GAL4-binding domain (BD) in a pGBKT7 vector. Yeast strain Y2HGold was transformed with the pGBKT7-M2 or pGBKT7-M2CT bait plasmid by using the lithium acetate method and was then mated with the Y187 yeast strain transformed with a pGADT7-based cDNA library prepared from a mixed human cell culture (A549, HEK293T, THP-1, and U251). Transformants were selected on medium that lacked adenine, histidine, leucine, and tryptophan (SD/−Ade/−His/−Leu/−Trp) (quadruple dropout medium [QDO]). Colonies were transferred to QDO plates containing 5-bromo-4-chloro-3-indolyl-α-d-galactopyranoside (X-α-Gal) and aureobasidin A (AbA) (SD/−Ade/−His/−Leu/−Trp/X-α-Gal/AbA) (QDO/X/A). Blue colonies were selected and cultured in medium that lacked leucine and tryptophan (SD/−Leu/−Trp) (DDO) for plasmid extraction. The plasmids were sequenced to identify the cellular interacting proteins. To confirm the interaction between M2 and the host proteins, the bait and prey plasmids were cotransformed into the Y2HGold yeast strain. Cotransformation of pGBKT7-53 encoding the Gal4 DNA-BD fused with murine p53 (BD-p53) and pGADT7-T encoding the Gal4 activation domain fused with the simian virus 40 large T antigen (AD-T) served as a positive control. Cotransformation of pGBKT7-Lam, which encodes the Gal4-BD fused with human lamin C (BD-Lam), and AD-T served as a negative control.

### Plasmids.

The TRAPPC6A gene was amplified by PCR from the plasmid recovered from the Y2H screen, and the TRAPPC6AΔ gene was amplified from cDNAs of A549 cells by reverse transcription-PCR amplification of total cellular mRNA with Superscript III reverse transcriptase (Invitrogen, Carlsbad, CA). These genes were subsequently cloned into the mammalian expression vector pCAGGS, resulting in constructs designated pCAGGS-TRAPPC6A and pCAGGS-TRAPPC6AΔ. Plasmids pCAGGS-TRAPPC6A-myc and pCAGGS-TRAPPC6AΔ-myc were generated by inserting the TRAPPC6A or TRAPPC6AΔ open reading frame (ORF) fused with the Myc tag sequence in the C terminus into the pCAGGS vector. The TRAPPC6A ORF was fused to the C terminus of GST in vector pGEX-6P-1 (GE Healthcare, Pittsburgh, PA), resulting in the construct pGEX-6P-1-TRAPPC6A. Plasmid pCAGGS-SC09M2 was constructed by inserting SC09M2 into pCAGGS. Plasmid pCAGGS-Flag-M2 was generated by inserting the M2 ORF fused with the Flag tag sequence in the N terminus into pCAGGS. The constructs pCAGGS-Flag-M2Del1, pCAGGS-Flag-M2Del2, pCAGGS-Flag-M2Del3, pCAGGS-Flag-M2Del4, pCAGGS-Flag-M2Del5, pCAGGS-Flag-M2Del6, pCAGGS-Flag-M2Del12, and pCAGGS-Flag-M2Del18 were created in the same way and expressed M2 with deletions of 1, 2, 3, 4, 5, 6, 12, and 18 residues from the C terminus, respectively. The pCAGGS-Flag-M2-96Mut plasmids, containing different amino acid mutations at position 96 of M2, were PCR amplified and cloned into pCAGGS. Plasmids pEGFP-C1-M2, pEGFP-C1-M2EDTM, and pEGFP-C1-M2CT were generated by inserting full-length M2 or truncated fragments into the pEGFP-C1 vector (Clontech). The BM2 sequence of B/Jilin/20/2003 virus (GenBank accession number CY033829.1) was synthesized (Comate Bioscience, Changchun, China) and inserted into the pEGFP-C1 vector to generate the construct pEGFP-C1-BM2. pRetroX-IRES-ZsGreen1-TRAPPC6AΔ was constructed by inserting the TRAPPC6AΔ ORF into the pRetroX-IRES-ZsGreen1 vector (Clontech). pHH21-M2Del1 and pHH21-M2Del2 were constructed by PCR amplification and subsequent cloning into the pHH21 vector as described previously (58); both constructs contained a stop codon at nucleotide positions 999 to 1001 or 1002 to 1004 of the M segment, resulting in the deletion of 1 and 2 residues from the C terminus of the M2 protein, respectively. All plasmid constructs were verified by sequencing. The primer sequences of all oligonucleotides used for cloning are available upon request.

### Antibodies.

The following primary antibodies were obtained from commercial sources: rabbit anti-Flag polyclonal antibody (F7425; Sigma-Aldrich), mouse anti-Flag monoclonal antibody (F3165; Sigma-Aldrich), rabbit anti-Myc polyclonal antibody (C3965; Sigma-Aldrich), mouse anti-Myc monoclonal antibody (M4439; Sigma-Aldrich), mouse anti-actin monoclonal antibody (sc-47778; Santa Cruz, Dallas, TX), rabbit anti-glyceraldehyde-3-phosphate dehydrogenase (GAPDH) polyclonal antibody (10494-1-AP; Proteintech, Chicago, IL), rabbit anti-GFP polyclonal antibody (AG279; Beyotime Biotech, Shanghai, China), mouse anti-GFP monoclonal antibody (ab1218; Abcam, Cambridge, MA), mouse anti-M2 monoclonal antibody (ab5416; Abcam), rabbit anti-M2 polyclonal antibody (GTX125951; GeneTex, Irvine, CA), rabbit anti-HA polyclonal antibody (11692-T54; Sino Biological Inc., Beijing, China), rabbit anti-FGF2 monoclonal antibody (ab92337; Abcam), mouse anti-LAMP1 monoclonal antibody (ab25630; Abcam), and mouse anti-Giantin monoclonal antibody (ab37266; Abcam). The rabbit anti-TRAPPC6A polyclonal antibody was made and stored in our laboratory. The commercially obtained secondary antibodies used in this study were DyLight 800 goat anti-mouse IgG(H+L) (072-07-18-06; KPL, Gaithersburg, MD), DyLight 800 goat anti-rabbit IgG(H+L) (072-07-15-06; KPL), Alexa Fluor 488 donkey anti-mouse IgG(H+L) (A21202; Life Technologies), Alexa Fluor 488 goat anti-rabbit IgG(H+L) (A11034; Life Technologies), and Alexa Fluor 546 donkey anti-rabbit IgG(H+L) (A10040; Life Technologies).

### Coimmunoprecipitation.

HEK293T cells cultured in 6-well plates were transfected with the respective plasmids (3 μg/each) by using Lipofectamine LTX and Plus reagents (Invitrogen) according to the manufacturer's instructions. At 48 h posttransfection, cells were washed three times with cold phosphate-buffered saline (PBS) (pH 7.4) and lysed with IP buffer (25 mM Tris-HCl [pH 7.4], 150 mM NaCl, 1% NP-40, 1 mM EDTA, 5% glycerol [Pierce, Rockford, IL]) containing a complete protease inhibitor cocktail (1 tablet/50 ml) (Roche Diagnostics GmbH, Mannheim, Germany) for 30 min on ice. The supernatants were then mixed and rocked at 4°C overnight with the indicated primary antibodies. Protein G-agarose beads (Roche) were added, and the mixture was rocked for 6 to 8 h at 4°C. The IgG-agarose beads were washed four times with 1 ml of wash buffer (25 mM Tris-HCl [pH 7.4], 150 mM NaCl, 1% NP-40), and immunoprecipitated proteins were separated by sodium dodecyl sulfate-polyacrylamide gel electrophoresis (SDS-PAGE) and detected by Western blotting.

### *In vitro* GST pulldown assay.

For an *in vitro* binding assay, GST-fused TRAPPC6A or GST alone was expressed in BL21(DE3) cells. The soluble fraction of the E. coli cell lysate was purified with glutathione-Sepharose 4B (GE Healthcare) and further fractionated by cation exchange chromatography. HEK293T cells transfected with 10 μg of the pCAGGS-SC09M2 or pCAGGS plasmid were washed twice with cold PBS and lysed with IP buffer. The lysate was split into two aliquots and separately incubated with 25 μg of purified GST-TRAPPC6A or GST for 2 h at 4°C. After three washes with IP buffer, the bound proteins were extracted from the Sepharose beads by boiling in 2× SDS loading buffer, separated by SDS-PAGE, and detected by Western blotting or Coomassie blue staining.

### Western blotting.

Proteins were resolved by SDS-PAGE and electrotransferred onto nitrocellulose membranes (GE Healthcare). The blots were blocked for 1 h at room temperature with 5% skim milk in a solution containing 137 mM NaCl, 2.7 mM KCl, 10 mM Na_2_HPO_4_, 2 mM KH_2_PO_4_, and 0.05% Tween 20 (PBST) and incubated overnight at 4°C with the appropriate primary antibodies diluted in PBST containing 2% BSA. After washing with PBST, the blots were incubated with DyLight 800 goat anti-mouse IgG(H+L) (1:10,000) or DyLight 800 goat anti-rabbit IgG(H+L) (1:10,000) and scanned by use of an Odyssey infrared imaging system (Li-Cor BioSciences, Lincoln, NE).

### siRNA transfection and virus infection.

A total of 2 × 10^5^ A549 cells were seeded into 12-well plates (Sigma-Aldrich) and then transfected with siRNA targeting TRAPPC6AΔ (5′-CUGUGUUGUUUGAGUUUCU-3′) or nontargeting siRNA (Genepharma, Shanghai, China) at a concentration of 40 nM for 48 h by using the Lipofectamine RNAiMAX transfection reagent (Invitrogen) according to the manufacturer's instructions. The knockdown efficiency was checked by Western blotting. To study the effect of TRAPPC6AΔ knockdown on the growth of influenza virus, the WSN virus was used to infect siRNA-treated A549 cells at an MOI of 0.01. Supernatants were collected at 24 and 48 h p.i., and virus titers were determined by plaque assays on MDCK cells.

### Cell viability assay.

Cell viability was determined by measuring the level of intracellular ATP with the CellTiter-Glo kit (Promega, Madison, WI) according to the manufacturer's instructions. Briefly, A549 cells seeded into opaque-walled 96-well plates were transfected with siRNA targeting TRAPPC6AΔ or with nontargeting siRNA at a concentration of 40 nM. At 48 h posttransfection, 100 μl of CellTiter-Glo reagent was added directly into each well, and the contents were mixed for 2 min on a shaker to induce cell lysis. After a 10-min incubation at room temperature, luminescence was recorded with a GloMax 96 microplate luminometer (Promega).

### Establishment of an A549 stable cell line overexpressing TRAPPC6AΔ and virus infection.

The pRetroX-IRES-ZsGreen1-TRAPPC6AΔ retroviral plasmid and an insertless pRetroX-IRES-ZsGreen1 control retroviral vector were independently transfected into the AmphoPack-293 packaging cell line (Clontech) by using Lipofectamine LTX and Plus reagents. Viral supernatants from transfectants were used to transduce A549 cells. After 48 h, transduction was repeated to enrich for transductants. GFP-positive cells were then harvested 48 h later by using a MoFlo XDP cell sorter (Beckman Coulter), propagated, and examined for TRAPPC6AΔ overexpression by Western blotting. To study the effect of TRAPPC6AΔ overexpression on the growth of influenza virus, the WSN virus was used to infect the TRAPPC6AΔ-overexpressing A549 cell line or an A549 control cell line transduced with empty retrovirus at an MOI of 0.01. Supernatants were collected at 24 and 48 h p.i., and virus titers were determined by plaque assays on MDCK cells.

### Generation of mutant influenza virus.

The mutant WSN viruses WSN M2Del1and WSN M2Del2 were generated from plasmids as described previously (58). Briefly, the eight plasmids for the synthesis of vRNA and the four protein expression plasmids for PB2, PB1, PA, and NP were transfected into HEK293T cells with Lipofectamine LTX and Plus reagents. Forty-eight hours later, the supernatant was harvested and stored at −80°C. Aliquots of the transfection supernatant were used to inoculate MDCK cells to produce stock viruses. The titers of the transfection supernatant and stock viruses were determined by plaque assays on MDCK cells. vRNA was extracted from the stock viruses by using the QIAamp viral RNA minikit (Qiagen, Valencia, CA). cDNA was synthesized from vRNA by reverse transcription-PCR with gene-specific primers. Sequencing was performed to confirm the identity of the mutant viruses.

### Growth curve analysis.

A549 cells (either untreated or treated with siRNA targeting TRAPPC6AΔ for 48 h as described above) were infected with the wt WSN virus or one of the mutant WSN viruses WSN M2Del1 and WSN M2Del2 at an MOI of 0.01. After 1 h of adsorption, removal of the inoculum, and one wash with F-12K medium (0.3% BSA), the cells were incubated with F-12K medium (0.3% BSA) at 37°C. The virus-containing culture supernatant was collected at various time points and titrated by plaque assays in MDCK cells.

### Confocal microscopy.

A549 cells grown in glass-bottom dishes were transiently transfected with the indicated plasmids by using Lipofectamine LTX and Plus reagents or were infected with WSN, WSN M2Del1, or WSN M2Del2 virus at an MOI of 5. At 24 h posttransfection or the indicated time points p.i., cells were washed with PBS, fixed with 4% paraformaldehyde (PFA) in PBS for 30 min at room temperature, and permeabilized with 0.5% Triton X-100 in PBS for 30 min. After such treatment, A549 cells were incubated with blocking buffer (5% BSA in PBS) for 1 h at room temperature. Primary antibodies (mouse anti-Flag monoclonal antibody, rabbit anti-Myc polyclonal antibody, mouse anti-M2 monoclonal antibody, rabbit anti-TRAPPC6A polyclonal antibody, rabbit anti-M2 polyclonal antibody, mouse anti-LAMP1 monoclonal antibody, or mouse anti-Giantin monoclonal antibody) were diluted in blocking buffer and incubated with cells for 2 h. The cells were then washed three times with PBS and incubated with secondary antibodies [Alexa Fluor 488 donkey anti-mouse IgG(H+L) and Alexa Fluor 546 donkey anti-rabbit IgG(H+L)] for 1 h. The cells were then washed three times with PBS and incubated with DAPI (4′,6-diamidino-2-phenylindole; Thermo Fisher Scientific, Waltham, MA) for 15 min. Cells were examined by using a Leica SP2 confocal system (Leica Microsystems, Wetzlar, Germany).

### Flow cytometry.

To examine whether the regulation of TRAPPC6AΔ expression affects the surface expression of M2, HA, or the cellular protein FGF2, the WSN virus was used to infect the indicated cells at an MOI of 3. The cells were collected at 8 and 10 h p.i. In an independent experiment, infected cells were subjected to an additional step of replacing the culture medium with medium supplemented with 25 μM amantadine (Sigma-Aldrich) at 2 h p.i. and then collected at 8 and 10 h p.i. The collected cells were fixed with 4% PFA for 30 min and then stained for the surface expression of M2, HA, or FGF2 with the corresponding primary and secondary antibodies [mouse anti-M2 MAb 14C2 and Alexa Fluor 488-conjugated donkey anti-mouse IgG(H+L) for M2, rabbit anti-HA polyclonal antibody and Alexa Fluor 488-conjugated goat anti-rabbit IgG(H+L) for HA, and rabbit anti-FGF2 MAb and Alexa Fluor 488-conjugated goat anti-rabbit IgG(H+L) for FGF2], without permeabilization treatment. After washing, the cell suspensions were subjected to flow cytometry on a FACSAria flow cytometer (BD Biosciences, Franklin Lakes, NJ). The data were analyzed by using FlowJo software.

### Animal experiments.

Six-week-old female BALB/c mice (Vital River Laboratories, Beijing, China) were lightly anesthetized with CO_2_ and intranasally infected with the wt WSN virus or the M2 deletion mutants WSN M2Del1 and WSN M2Del2 in 50 μl of PBS, with doses ranging from 10^3^ to 10^6^ PFU per mouse. Four mice were infected with each dose and observed for 14 days p.i., and their body weight and survival status were checked daily.

### Plaque assay.

Plaque assays were performed on MDCK cells in 12-well plates according to standard procedures. Briefly, cells were infected with serial 10-fold dilutions of the virus supernatants in 1× MEM (0.3% BSA) for 1 h at 37°C. The inoculum was removed, and the cells were washed with PBS and overlaid with 1% SeaPlaque agarose (Lonza, Rockland, ME) in 1× MEM (0.3% BSA, 0.5 μg/ml TPCK-treated trypsin). After 2 to 3 days of incubation, the cells were fixed with formalin in PBS, and plaques were counted.

### Statistical analysis.

Results were analyzed for statistical significance by using Student's *t* test. A *P* value of <0.05 was considered statistically significant.
